# Methotrexate and Tumor Necrosis Factor Inhibitors Independently Decrease Neutralizing Antibodies after SARS-CoV-2 Vaccination: Updated Results from the SUCCEED Study

**DOI:** 10.3390/vaccines12091061

**Published:** 2024-09-17

**Authors:** Carol A Hitchon, Dawn M. E. Bowdish, Gilles Boire, Paul R. Fortin, Louis Flamand, Vinod Chandran, Roya M. Dayam, Anne-Claude Gingras, Catherine M. Card, Inés Colmegna, Maggie J. Larché, Gilaad G. Kaplan, Luck Lukusa, Jennifer L.F. Lee, Sasha Bernatsky, on behalf of the SUCCEED Investigative Team

**Affiliations:** 1 Department of Internal Medicine, Max Rady College of Medicine, Rady Faculty of Health Sciences, University of Manitoba, Winnipeg, MB R3T 2N2, Canada; 2 McMaster Immunology Research Centre, McMaster University, Hamilton, ON L8S 4L8, Canada; 3 Division of Rheumatology, Department of Medicine, Université de Sherbrooke, Sherbrooke, QC J1H 5N4, Canada; 4 Centre de Recherche ARThrite—UL, Division of Rheumatology, Department of Medicine, CHU de Québec, Université Laval, Québec City, QC G1V 4G2, Canada; 5 Axe Maladies Infectieuses et Immunitaires, Centre de Recherche du CHU de Québec, Université Laval, Québec City, QC G1V 4G2, Canada; 6 Schroeder Arthritis Institute and Krembil Research Institute, University Health Network, Toronto, ON M5T 2S8, Canada; 7 Lunenfeld-Tanenbaum Research Institute at Mount Sinai Hospital, Sinai Health, Toronto, ON M5G 1X5, Canada; 8 Department of Medical Microbiology and Infectious Diseases, University of Manitoba, Winnipeg, MB R3T 2N2, Canada; 9 JC Wilt Infectious Diseases Research Centre, National Microbiology Laboratory, Public Health Agency of Canada, Winnipeg, MB R3T 2N2, Canada; 10 Department of Medicine, McGill University, Montreal, QC H3A 0E9, Canada; ines.colmegna.med@ssss.gouv.qc.ca; 11 The Research Institute of the McGill University Health Centre, McGill University, Montreal, QC H3A 0E9, Canada; 12 Division of Rheumatology, Department of Medicine, McMaster University, Hamilton, ON L8S 4L8, Canada; 13 Division of Gastroenterology and Hepatology, Departments of Medicine and Community Health Sciences, University of Calgary, Calgary, AB T2N 1N4, Canada; ggkaplan@ucalgary.ca

**Keywords:** COVID-19, vaccination, immune-mediated inflammatory disease, methotrexate, tumor necrosis factor inhibitors, autoimmune diseases, TNF-blocking antibody

## Abstract

**Objective:** SARS-CoV-2 remains the third most common cause of death in North America. We studied the effects of methotrexate and tumor necrosis factor inhibitor (TNFi) on neutralization responses after COVID-19 vaccination in immune-mediated inflammatory disease (IMID). **Methods:** Prospective data and sera of adults with inflammatory bowel disease (IBD), rheumatoid arthritis (RA), spondyloarthritis (SpA), psoriatic arthritis (PsA), and systemic lupus (SLE) were collected at six academic centers in Alberta, Manitoba, Ontario, and Quebec between 2022 and 2023. Sera from two time points were evaluated for each subject. Neutralization studies were divided between five laboratories, and each lab’s results were analyzed separately using multivariate generalized logit models (ordinal outcomes: absent, low, medium, and high neutralization). Odds ratios (ORs) for the effects of methotrexate and TNFi were adjusted for demographics, IMID, other biologics and immunosuppressives, prednisone, COVID-19 vaccinations (number/type), and infections in the 6 months prior to sampling. The adjusted ORs for methotrexate and TNFi were then pooled in random-effects meta-analyses (separately for the ancestral strains and the Omicron BA1 and BA5 strains). **Results:** Of 479 individuals (958 samples), 292 (61%) were IBD, 141 (29.4%) were RA, and the remainder were PsA, SpA, and SLE. The mean age was 57 (62.2% female). For both the individual labs and the meta-analyses, the adjusted ORs suggested independent negative effects of TNFi and methotrexate on neutralization. The meta-analysis adjusted ORs for TNFi were 0.56 (95% confidence interval (CI) 0.39, 0.81) for the ancestral strain and 0.56 (95% CI 0.39, 0.81) for BA5. The meta-analysis adjusted OR for methotrexate was 0.39 (95% CI 0.19, 0.76) for BA1. **Conclusions:** SARS-CoV-2 neutralization in vaccinated IMID was diminished independently by TNFi and methotrexate. As SARS-CoV-2 circulation continues, ongoing vigilance regarding optimized vaccination is required.

## 1. Introduction

In North America, COVID-19 continues to be the third most common cause of death, right after cancer and heart disease [1]. Given the ongoing SARS-CoV-2 circulation, the COVID-19 vaccination response in people with immune-mediated inflammatory disease (IMID) remains a key issue, particularly regarding the effects of common immunosuppressives like methotrexate and tumor necrosis factor inhibitors (TNFi). To date, most studies have focused on a single disease and/or a single center or assessed only ancestral strains and/or only the presence of antibodies and not viral neutralization ability. When combining data across centers to achieve a larger and more diverse sample, sophisticated modeling must allow for differences across centers while aiming to achieve a single estimate of effects. Our purpose was to overcome these challenges.

## 2. Methods

In this prospective observational multicenter cohort study, we recruited adults with inflammatory bowel disease (IBD), psoriatic arthritis (PsA), rheumatoid arthritis (RA), spondylarthritis (SpA), and systemic lupus erythematosus (SLE) from participating tertiary care rheumatology centers across Canada (from Calgary, Winnipeg, Quebec City, Sherbrooke, Toronto, and Hamilton). Ethics approval was obtained from the McGill University Health Centre and participating centers, and participants provided written or oral consent. Initial recruitment began early in 2021, shortly after Canada began vaccinating against SARS-CoV-2 (mostly with mRNA formulations), and continued until the end of 2022. We required only a physician diagnosis of IMID, not specific diagnostic criteria. Exclusion criteria were individuals who could not provide consent in English or French and those whose last COVID-19 vaccination was over 6 months prior to recruitment (unless they planned to be vaccinated in the near future). The current analyses focused on neutralizing antibodies detected in paired sera (mostly after the 3rd or 4th vaccination).

Participants provided baseline and follow-up information on demographics (current age, sex at birth, and self-identified race/ethnicity according to categories), past COVID-19 infections (any confirmed event reported by subjects), COVID-19 vaccinations (including dates and type), and clinical history (type of IMID, date of diagnosis, and medications). Neutralization studies are time- and resource-intensive. Since the capacity across the labs that supported multiple clinical and research studies was limited, the analysis was performed across multiple laboratories. The sera from Toronto, Sherbrooke and some Winnipeg samples were assessed in the Gingras laboratory for neutralization using a lentivirus-based spike pseudotype assay of three SARS-CoV-2 strains, as described previously [2]. Samples from Hamilton, Quebec City, Calgary and some samples from Winnipeg were processed locally, as per Table 1. All labs (Toronto, Hamilton [3], Quebec City [4], Calgary [5], and Winnipeg [6]) performed neutralization for the ancestral strain; four of these labs (those in Winnipeg, Quebec City, Toronto, and Hamilton) also assessed Omicron BA1, and three (in Winnipeg, Quebec City, and Toronto) also assessed Omicron BA5.

Two serum samples from each participant collected between 2022 and 2023 were assessed. We required samples to be at least 30 days beyond the last vaccination. Each lab’s results were analyzed separately using 3 multivariate generalized logit models for the ancestral, Omicron BA1, and BA5 strains, with ordinal outcomes for no, low, medium, and high neutralization, as detailed in Table 1. Then, the results were pooled across labs in random-effects meta-analyses (separately for the ancestral, BA1, and BA5 strains). This approach allowed for differences in effect sizes since methods differed between labs. As an exploration/validation study, the Toronto (Gingras) lab also received and analyzed 10 random samples from each of the other centers.

In all our models for the primary analyses, potential predictors, effect modifiers, and/or confounders included demographics (age, biologic sex, and self-reported race/ethnicity), COVID-19 vaccinations (timing and type), reports of SARS-CoV-2 infection (all lab-confirmed events reported by the subject within 6 months of sample), and clinical factors, such as the IMID type and current (at our study enrolment) medication use. Besides methotrexate and TNFi, we adjusted for other disease-modifying agents (disease-modifying antirheumatic drugs (DMARDs), including azathioprine, sulfasalazine, leflunomide, Janus kinase inhibitors (JAKi), and 6-mercaptopurine), other biologics (ustekinumab, vedolizumab, abatacept, rituximab, tocilizumab, secukinumab) and prednisone. All medication variables in the model reflected current use at enrolment (yes or no) as per Table 2.

The vaccine history variables in our models included the number of vaccines at the time of sampling (dichotomous variable: 2–3 vs. 4+ doses) and type (categorical for BNT162b2 monovalent only, mRNA1273 monovalent only, any combination of these monovalent pre-Omicron vaccines, any bivalent formulations targeting Omicron, and other), as per Table 2. We also controlled for days between the last vaccine and the sample (dichotomized at 120+ days post-vaccine versus earlier) and whether or not the individual reported a positive test for COVID-19 infection in the 6 months prior to sample collection.

Statistical analyses were performed with R software (Version R 4.3.3). We used the “repolr” function of the GEE package for our ordinal outcomes analyses and the “rma” function of the metafor package for our random-effects meta-analyses.

## 3. Results

We studied 479 individuals; of these, 292 (61%) had IBD, 141 (29.4%) RA, 24 (5%) PsA, 13 (2.7%) SpA, and 9 (1.9%) SLE. Most individuals (447, 93.3%) were white, 62.2% were female, and the mean age was 56.8 (standard deviation 14.8) years. The other characteristics are shown in Table 2.

About 20% of individuals were on prednisone upon study entry, most below 10 mg per day (Table 2). In terms of biologics, 186 (38.8% of the 479 individuals) were on a TNFi, 80 (16.7%) were on ustekinumab, 47 (9.8%) were on vedolizumab, 15 (3.1%) were on abatacept, and 8 (1.7%) were on rituximab. Of the 186 TNFi users, 79 were on adalimumab, 78 were on infliximab, 20 were on etanercept, 7 were on golimumab, and 2 were on certolizumab.

Regarding DMARDs, 122 (25.5% of the 479 individuals) were on methotrexate, 29 (6.1%) were on sulfasalazine, 24 (5%) were on azathioprine, 20 (4.2%) were on leflunomide, and 18 (3.8%) were on a JAKi. A number (82, 17.1%) were on hydroxychloroquine. However, we did not include these in the DMARD category given that hydroxychloroquine does not have the same effects on suppressing antibody formation after vaccination that other DMARDs may have [7].

At first sample, about 40% of subjects had more than two vaccinations, while in the second sample, this had increased to almost 70%. About two-thirds of participants had received BNT162b2 monovalent only prior to providing a sample. The majority (about 85%) of samples were provided by individuals who had not received an Omicron-targeted vaccine formulation. Thirty-four (7.1%) of individuals providing samples reported clinically confirmed COVID-19 infection in the 6 months prior to collection of both the first and second samples.

Table 3 shows the odds ratios (ORs) for neutralization related to methotrexate and to TNFi, adjusted for sex, age, race/ethnicity, IMID, DMARDs, biologics, prednisone, and the details of past COVID-19 vaccinations and infection. For both the individual labs and the meta-analyses, the adjusted ORs suggested that TNFi and methotrexate were independently associated with a lower neutralization ability. The meta-analysis adjusted ORs for TNFi were 0.56 (95% confidence interval (CI) 0.39, 0.81) for the ancestral strain and 0.56 (95% CI 0.39, 0.81) for Omicron BA5. The meta-analysis adjusted OR for methotrexate was 0.39 (95% CI 0.19, 0.76) for Omicron BA1.

The Supplemental Tables show the results for individual models related to each strain and lab. Across these models, the effects of demographics, medications, and vaccine history were largely similar.

## 4. Discussion

Neutralizing antibodies against the SARS-CoV-2 inhibit the virus’s ability to enter human cells and likely play a key role in protecting an individual from COVID-19 infection and severity [8]. Our results indicate that neutralization responses in immunosuppressed IMID hosts may be diminished by both TNFi and methotrexate in an independent manner.

Methotrexate is a ‘cornerstone’ DMARD in RA and is useful in SLE and other autoimmune diseases. It is a cytostatic agent that limits the synthesis of purine nucleotides and their derivatives, diminishing the proliferation of B cells and T cells. It thus has the potential to lower antibody production not only in rheumatic disease but also after vaccination. Given the importance of this drug, several advisory groups have sought to provide recommendations to patients and healthcare providers, such as holding methotrexate (and/or some biologics) around the time of COVID-19 vaccination. However, individuals may be concerned that this might trigger IMID symptoms. In a recent small meta-analysis, withholding MTX for approximately 2 weeks following COVID-19 vaccination, though associated with significantly higher antibody titers, was also associated with a higher disease flare rate [9]. A potential limitation of our study is that we did not include information on whether patients continued or held their medications (and for how long) in our analyses.

TNF inhibitors have also been reported to lower concentrations of anti-receptor binding domain (and other anti-spike antibodies), increase decay over time in these antibodies, and increase the occurrence of breakthrough infections [10,11,12]. However, most prior studies collected data on a single disease (e.g., IBD or RA) and/or focused on a single drug class (e.g., anti-TNFi), either excluding those with other concomitant medications (including prednisone) or not adjusting for them. In the real world, decisions have to be made across a broad range of IMID types and concomitant medications. Our data thus add to the existing literature; specifically, our study is the first detailed analysis of the independent effects of methotrexate and TNFi on neutralizing antibodies in a broad range of IMID individuals that accounts for both past infection and detailed vaccination history.

The work of Garner-Spitzer et al. suggested that in anti-TNF-α-exposed individuals who have been vaccinated, diminished neutralization capacity against SARS-CoV-2 may be related to the impaired formation and maintenance of specific B memory cells, likely due to absent circulating T follicular helper cell activation, affecting extra-follicular immune responses and diminishing B memory cell diversification [13].

In many countries, including Canada and the United States, although two mRNA vaccines represent a primary series for immunocompetent people [14], three doses are considered the primary series in individuals receiving immunosuppressives, according to Centre for Disease Control, CDC, Public Health Agency of Canada, and ACR guidelines. These guidelines additionally point out that in individuals taking IMID medications that impair vaccine responsiveness, supplemental doses are recommended (e.g., ≥2 additional boosters for a total of five doses, as per the ACR and CDC guidelines).

The strengths of our work are that we studied a large group of individuals from multiple centers with several diseases who were on a range of different therapies, which reflects how complicated IMID care is. We had multiple samples and detailed, longitudinal information on vaccine and infection history. The potential limitations include the complex nature of this real-world dataset, particularly with respect to the logistical challenges of decentralized analysis, which resulted in the heterogeneity of approaches used by the different labs to assess neutralization. However, we were able to see similar effects of our demographic and clinical variables across the different methods, and to acknowledge the differences between laboratories, we modeled the data separately and then performed a meta-analysis to produce overall estimates for the effects of methotrexate and TNFi.

Another strength of our study is that we controlled all estimates for concomitant use of a variety of medications. However, our current approach did simplify medication exposures in that we modeled medications at cohort entry only. Also, in reality, higher doses of these drugs may have a more pronounced effect on the immune response than lower doses. Fortunately, our data spanned a relatively short time period, and medications taken were relatively stable over time in the individuals that we studied. Though we asked subjects about whether they held their medications at the time of vaccination, this information ended up being difficult to include in our modeling because it is so complex. For example, most participants did not hold their methotrexate before their vaccine doses, but a few held the methotrexate for one (or two) weeks prior to and not after or for one or two weeks after and not before. Altogether, the data we had were not easy to integrate into the current analyses.

Even with almost 500 patients and almost 1000 samples, however, we did have relatively small numbers of individuals on specific medications of potential interest (e.g., we only had eight individuals on rituximab), allowing us to adjust for these medications but precluding precise estimates of all drug effects.

Since an objective of our study was to capture the breadth of the general IMID population, we aimed to use as few exclusion criteria as possible (i.e., individuals with IMID were clinically confirmed, but we did not require specific diagnostic criteria or medication exposures). It remains impossible to exclude some selection bias, and the age and sex characteristics of our sample are largely similar to what was expected. Race/ethnicity diversity was somewhat lacking in that over 93% of participants self-reported as white; however, many of the centers from which people were recruited were white-predominant (including Quebec City and Sherbrooke, which are over 90% white). Non-white Canadians may be less likely to be vaccinated against preventable diseases, including COVID-19 [15].

Otherwise, potential limitations or criticisms of our study may include that there is no comparison with individuals who do not have IMID; and that the design of the study did not allow us to exclude the influence of pathogenetic factors related to IMID itself. The most important potential limitation is that we did not study the detailed effects of the dose and duration of the immunosuppressive therapies that our participants were receiving. Although we found negative associations between MTX and TNFi use and reduced neutralizing antibody responses, our findings remain observational.

In summary, our detailed analyses of neutralization ability post-COVID-19 vaccination in IMID suggest independent effects of methotrexate and TNFi. This reminder is important given the stark reality of vaccine hesitancy, even among immunocompromised individuals [16]. Given the ongoing waves of SARS-CoV2 circulation (including new variants), people with IMID, and their caregivers should remain aware of the need to optimize vaccine coverage against SARS-CoV-2.

## Figures and Tables

**Table 1:** Information on the laboratories conducting COVID-19 neutralization assays.

Location N	Variants	Result				Units Reported	Description
		Negative	Low Positive	Medium Positive	High Positive		
Calgary 246	Ancestral	<20	20–200	200–1620	>1620	50% neutralization titer (NT50).	Surrogate-vesicular stomatitis virus plaque reduction neutralization test (PRNT)
Gingras Lab, Toronto 116	Ancestral, Omicron, BA.1, BA.5	<1.5 (<32)	1.5–2 (32–100)	2–3 (100–1000)	>3 (>1000)	Log_10_ ID50 (ID50 is the dilution at which 50% neutralization occurs)	Spike-pseudotyped lentivirus neutralization
Bowdish Lab, Hamilton 50	Ancestral, Omicron BA.1	<=5	10–160	329–640	1280	Highest dilution achieving geometric microneutralization of 50% (MNT50)	Cell culture assays with live SARS-CoV-2
Card Lab, Winnipeg 35	Ancestral, Omicron BA.1, BA.5	<40 %inhibition	40–69.9%	70–89.9%	>90% inhibition	% inhibition	Surrogate nAb analysis using the MSD platform. Kit: V-PLEX SARS-CoV-2 Key Variant
Flamand Lab, Quebec City 30	Wuhan, Omicron BA.1, BA.5	<20	20–200	200–1620	>1620	Highest serum dilution preventing infection (100% neutralization)	Live-virus SARS-CoV-2 neutralization

**Table 2:** SUCCEED participants contributing samples for neutralization assays.

Variables	N = 479	
Province, N (%)		
Alberta (Calgary)	257 (53.7)	
Manitoba	90 (18.8)	
Ontario	73 (15.2)	
Quebec	59 (12.3)	
Mean days between samples (standard deviation, SD)	97.2 (50.8)	
Mean IMID duration at first/second sample (SD), years	18.9 (14.4)	
Baseline prednisone, N (%)	92 (19.2)	
Baseline prednisone dose, N (%)		
1–10 mg	58 (12.1)	
11–20 mg	9 (1.9)	
20+ mg	24 (5.0)	
Missing dose	1 (0.2)	
Baseline biologic, N (%)		
Tumor necrosis factor inhibitor	186 (38.8)	
Ustekinumab	80 (16.7)	
Vedolizumab	47 (9.8)	
Abatacept	15 (3.1)	
Rituximab	8 (1.7)	
Other biologics ^a^	8 (1.7)	
Baseline non-biologic drugs, N (%)		
Methotrexate	122 (25.5)	
Azathioprine	24 (5.0)	
Sulfasalazine	29 (6.1)	
Leflunomide	20 (4.2)	
JAK inhibitor	18 (3.8)	
6-Mercaptopurine	1 (0.2)	
^a^ Other biologics included tocilizumab and secukinumab		
**Variables**	**First sample**	**Second sample**
Vaccine doses before the sample N (%)		
Two	288 (60.1)	147 (30.8)
Three	110 (23.0)	216 (45.0)
Four	40 (8.4)	67 (14.0)
Five or more	41 (8.6)	49 (10.2)
Vaccine type		
BNT-162b2 monovalent only	313 (65.3)	302 (63.0)
Mixed bivalent	65 (13.6)	72 (15.1)
Mixed monovalent	61 (12.7)	69 (14.4)
mRNA1273 monovalent	33 (6.9)	30 (6.3)
Other	7 (1.5)	6 (1.3)
Mean days between last vaccine and sample (SD)	38.5 (33.7)	87.6 (57.3)
Calendar year ≥ 2022, N (%)	109 (22.8)	161 (33.6)
Calendar period N (%)		
April to Sept	305 (63.6)	171 (35.7)
Oct to March	174 (36.3)	308 (64.1)

**Table 3:** Multivariate ordered logit regression ^a^ and random-effects meta-analyses ^b^: Adjusted odds ratios (aORs) for the effects of methotrexate and tumor-necrosis factor inhibitor (TNFi) on neutralization with 95% confidence intervals (CIs).

SARS-Cov2 Strain	Number of Subjects	Methotrexate		TNFi	
		aOR	95% CI	aOR	95% CI
**Ancestral**	N = 116 Gingras	**0.25**	**(0.11, 0.56)**	0.74	(0.31, 1.79)
	N = 30 Flamand	0.51	(0.01, 38.7)	0.79	(0.19, 3.11)
	N = 35 Card	**0.04**	**(0.01, 0.22)**	0.29	(0.07, 1.15)
	N = 50 Bowdish	2.55	(0.77, 8.99)	1.53	(0.40, 6.05)
	N = 248 Calgary	0.64	(0.36, 1.14)	**0.48**	**(0.30, 0.75)**
	**Meta-analysis ^b^**	0.41	(0.10, 1.61)	**0.56**	**(0.39, 0.81)**
**Omicron BA1**	N = 116 Gingras	**0.29**	**(0.14, 0.59)**	1.11	(0.54, 2.27)
	N = 30 Flamand	0.41	(0.05, 3.14)	**0.04**	**(0.01, 0.27)**
	N = 35 Card	0.11	(0.01, 1.44)	0.59	(0.03, 6.79)
	N = 50 Bowdish	0.80	(0.26, 2.44)	0.29	(0.07, 1.08)
	**Meta-analysis ^b^**	**0.39**	**(0.19, 0.76)**	0.35	(0.09, 1.39)
**Omicron BA5**	N = 116 Gingras	**0.33**	**(0.16, 0.67)**	0.73	(0.35, 1.51)
	N = 30 Flamand	2.02	(0.27, 17.1)	**0.06**	**(0.01, 0.29)**
	N = 35 Card	0.50	(0.09, 2.67)	**0.08**	**(0.01, 0.39)**
	**Meta-analysis ^b^**	**0.48**	**(0.20, 1.13)**	**0.18**	**(0.03, 0.95)**

^a^ Repeated measures ordered logit generalized estimating equation regression adjusting for sex, age at sampling, race/ethnicity, IMID type, other biologics and non-biologic immunosuppressives, prednisone, COVID-19 infection in the 6 months prior to sampling, and number/type of COVID-19 vaccinations. The level of outcome was negative, low, medium, or high neutralization. ^b^ Random effects meta-analyses of adjusted ORs. Bold represents a significant value.

## Data Availability

The data are contained within the article. The original contributions presented in the study are included in the article; further inquiries can be directed to the corresponding author.

## Supplementary Materials

The following supporting information can be downloaded at: https://www.mdpi.com/article/10.3390/vaccines12091061/s1, Table S1: Gingras Lab Generalized Estimating Equation Logistic Regression, Crude and adjusted Odds Ratios (Ordinal Scores); Table S2: Calgary Lab: Generalized Estimating Equation Logistic Regression, Crude and adjusted Odds Ratios (Ordinal Scores); Table S3a: Flamand Lab: Generalized Estimating Equation Logistic Regression, Crude and adjusted Odds Ratios (Ordinal Scores); Table S3b: Flamand Lab: Generalized Estimating Equation Logistic Regression, Crude and adjusted Odds Ratios (Ordinal Scores); Table S4a: Card Lab: Generalized Estimating Equation Logistic Regression, Crude and adjusted Odds Ratios (Ordinal Scores); Table S4b: Card Lab: Generalized Estimating Equation Logistic Regression, Crude and adjusted Odds Ratios (Ordinal Scores); Table S5: Bowdish Lab: Generalized Estimating Equation Logistic Regression, Crude and adjusted Odds Ratios (Ordinal Scores).
