# Supplementary material for: Methotrexate and Tumor Necrosis Factor Inhibitors Independently Decrease Neutralizing Antibodies after SARS-CoV-2 Vaccination: Updated Results from the SUCCEED Study
Source: Vaccines (Basel). 2024 Sep 17;12(9):1061. doi: 10.3390/vaccines12091061 (PMC11436066; doi:10.3390/vaccines12091061)
Supplement: Supplementary file 1 [file vaccines-12-01061-s001.zip › vaccines-3172823-supplementary.pdf]

SUPPLEMENTAL TABLES- Individual lab results

Table S1 Gingras Lab Generalized Estimating Equation Logistic Regression, Crude and adjusted Odds Ratios (Ordinal Scores)

Characteristics	Ancestral variant		Omicron BA1 variant		BA5 variant	
	Crude OR (95% CI)	Adjusted OR (95% CI)[a]	Crude OR (95% CI)	Adjusted OR (95% CI)[a]	Crude OR (95% CI)	Adjusted OR (95% CI)[a]
Female sex	1.03 (0.57, 1.85)	1.49 (0.71, 3.11)	0.62 (0.38, 1.02)	0.68 (0.37, 1.26)	0.84 (0.52, 1.38)	1.22 (0.67, 2.23)
Age at sample, years	0.98 (0.73, 1.31)	0.88 (0.59, 1.29)	1.44 (1.12, 1.88)	1.16 (0.84, 1.62)	1.16 (0.91, 1.47)	0.81 (0.59, 1.11)
White race_ethnicity	1.66 (0.71, 3.73)	1.64 (0.56, 4.68)	2.06 (1, 4.34)	1.51 (0.63, 3.67)	2.74 (1.32, 5.84)	3.09 (1.27, 7.74)
Imid type IBD	1 (0.45, 2.18)	1.73 (0.61, 4.95)	0.71 (0.36, 1.39)	2.63 (1.05, 6.74)	0.7 (0.37, 1.32)	3.05 (1.26, 7.49)
Imid type RA	1.25 (0.61, 2.51)	3.29 (1.17, 9.52)	1.12 (0.6, 2.11)	3.3 (1.36, 8.35)	0.98 (0.53, 1.81)	2.73 (1.12, 6.85)
Vaccine type : Moderna mono	2.51 (0.58, 17.4)	1.99 (0.37, 15.9)	2.88 (0.87, 9.61)	1.78 (0.45, 7.1)	1.65 (0.48, 5.51)	1.24 (0.31, 4.88)
Vaccine type : Mixed mono	1.57 (0.79, 3.21)	1.67 (0.75, 3.81)	1.2 (0.64, 2.24)	1.11 (0.54, 2.28)	0.91 (0.49, 1.68)	0.83 (0.41, 1.67)
Vaccine type : Mixed bivalent	7.32 (2.71, 25.65)	9.35 (2.71, 39.7)	3.19 (1.39, 7.83)	4.03 (1.43, 12.3)	4.53 (1.74, 14.32)	6.60 (2.09, 24.90)
Number of vaccine :Three or more	2.52 (1.41, 4.55)	1.43 (0.67, 3.04)	4.03 (2.42, 6.8)	1.95 (1.02, 3.79)	3.53 (2.14, 5.87)	1.82 (0.96, 3.46)
Number days : 121 + days	1.03 (0.44, 2.63)	0.73 (0.25, 2.27)	1.81 (0.86, 3.84)	1.07 (0.42, 2.68)	2.44 (1.17, 5.22)	1.66 (0.63, 4.46)
Prednisone	0.65 (0.31, 1.43)	0.4 (0.16, 1.04)	1.2 (0.61, 2.35)	0.6 (0.26, 1.33)	1.39 (0.71, 2.76)	0.89 (0.38, 2.09)
Anti-TNF biologic	0.85 (0.43, 1.74)	0.74 (0.31, 1.79)	0.79 (0.43, 1.41)	1.11 (0.54, 2.27)	0.72 (0.4, 1.29)	0.73 (0.35, 1.51)
Ustekinumab	1.15 (0.37, 4.33)	1.17 (0.25, 6.3)	1.19 (0.48, 2.89)	0.95 (0.27, 3.2)	1.36 (0.53, 3.56)	0.93 (0.28, 3.19)
Rituximab	0.28 (0.08, 0.96)	0.61 (0.13, 3.13)	0.46 (0.12, 1.6)	0.41 (0.06, 2.31)	0.48 (0.13, 1.65)	0.38 (0.05, 2.17)
Other biologic	0.84 (0.28, 2.81)	0.95 (0.23, 4.76)	1.83 (0.76, 4.45)	2.01 (0.68, 6.02)	1.78 (0.65, 5.02)	2.6 (0.71, 9.72)
Methotrexate	0.41 (0.23, 0.74)	0.25 (0.11, 0.56)	0.53 (0.32, 0.89)	0.29 (0.14, 0.59)	0.51 (0.3, 0.85)	0.33 (0.16, 0.67)
Jak inhibitor	0.89 (0.27, 3.45)	2.46 (0.35, 19.7)	1.41 (0.47, 4.4)	6.9 (1.25, 41.77)	1.85 (0.61, 5.99)	11.2 (1.85, 75.57)
Other immuno	0.85 (0.42, 1.77)	0.81 (0.3, 2.27)	0.71 (0.36, 1.37)	0.45 (0.18, 1.12)	0.65 (0.34, 1.24)	0.42 (0.17, 1.04)
Past infection in the 6 months before sample	3.78 (0.66, 71.25)	4.85 (0.75, 95.9)	4.66 (1.28, 19.12)	8.92 (1.77, 49.9)	4.77 (1.23, 23.27)	19.3 (3.52, 124.1)

[a] Adjusted for all variables shown.. [c] Other biologic included : abatacept, Vedolizumab , Tocilizumab and Secukinumab.. [d] Other immuno included : azathioprine, 6-mercaptopurine, leflunomide, and sulfasalazine. Here the reference for the number of vaccines was 2 doses. Here the reference for IMID type was "SLE, PsA,SpA".

SUPPLEMENTAL TABLES- Individual lab results

Table S2 Calgary Lab:* *Generalized Estimating Equation Logistic Regression, Crude and adjusted Odds Ratios (Ordinal Scores)

**Characteristics**	**Ancestral variant**	
	**Crude OR (95% CI)**	**Adjusted OR (95% CI)[a]**
Female sex	0.96 (0.69, 1.32)	1.02 (0.72, 1.45)
Age at sample, years	0.93 (0.79, 1.09)	0.86 (0.72, 1.03)
White race_ethnicity	1.00 (0.50, 2.03)	0.99 (0.48, 2.12)
Vaccine type : Moderna mono	1.18 (0.73, 1.90)	1.27 (0.74, 2.13)
Vaccine type : Mixed mono	1.84 (0.91, 3.69)	**2.37 (1.15, 4.83)**
Number of vaccine :Three or more	5.33 (0.50, 59.7)	1.10 (0.06, 19.9)
Number days : 121 + days	**0.2 (0.11, 0.34)**	**0.16 (0.09, 0.29)**
Prednisone	0.80 (0.42, 1.50)	0.92 (0.45, 1.82)
Anti-TNF biologic	**0.36 (0.26, 0.5)**	**0.48 (0.30, 0.75)**
Ustekinumab	1.82 (1.27, 2.62)	1.57 (0.97, 2.53)
Other biologic	**2.7 (1.72, 4.25)**	**2.75 (1.54, 4.93)**
Methotrexate	0.70 (0.41, 1.17)	0.64 (0.36, 1.14)
Jak inhibitor	0.76 (0.15, 3.08)	0.58 (0.10, 2.65)
Other immuno	0.75 (0.44, 1.25)	0.66 (0.35, 1.19)
Past infection in the 6 months before sample	**5.66 (3.08, 10.5)**	**5.99 (3.18, 11.4)**

[a] Adjusted for all variables shown.. [c] Other biologic included : abatacept, Vedolizumab , Tocilizumab and Secukinumab.. [d] Other immuno included : azathioprine, 6-mercaptopurine, leflunomide, and sulfasalazine. Here the reference for the number of vaccines was 2 doses. Here, all patients had IBD and no patient was exposed to Rituximab. None had bivalent vaccine

SUPPLEMENTAL TABLES- Individual lab results

Table S3a: Flamand Lab: Generalized Estimating Equation Logistic Regression, Crude and adjusted Odds Ratios (Ordinal Scores)

Characteristics	Ancestral variant	
	Crude OR (95% CI)	Adjusted OR (95% CI)[a]
Female sex	0.8 (0.27, 2.37)	0.52 (0.12, 2.20)
Age at sample, years	0.69 (0.41, 1.13)	0.62 (0.29, 1.27)
Imid type RA	0.62 (0.22, 1.67)	0.52 (0.01, 30.5)
Number of vaccine :Four or more	0.5 (0.01, 19.12)	1.37 (0.02, 86.7)
Number days : 121 + days	0.9 (0.21, 3.92)	0.55 (0.06, 4.54)
Prednisone	0.58 (0.21, 1.58)	1.33 (0.26, 6.96)
Anti-TNF biologic	0.81 (0.26, 2.58)	0.79 (0.19, 3.11)
Other biologic	2.1 (0.35, 13.15)	0.53 (0.01, 63.1)
Methotrexate	0.29 (0.1, 0.81)	0.51 (0.01, 38.7)
Jak inhibitor	2.1 (0.35, 13.15)	0.77 (0.02, 33.4)
Other immuno	4.77 (1.37, 18.87)	9.16 (0.09, 123)
Past infection in the 6 months before sample	1.63 (0.55, 4.98)	0.70 (0.13, 3.56)

[a] Adjusted for all variables shown.. [c] Other biologic included : abatacept, Vedolizumab , Tocilizumab and Secukinumab.. [d] Other immuno included : azathioprine, 6-mercaptopurine, leflunomide, and sulfasalazine. Here the reference for the number of vaccines was 3 doses. Here the reference for IMID type was "SpA".
* Here the reference for vaccine type was "Pfizer monovalent". No one was exposed to rituximab and everyone was white.
**I removed the type of vaccine, type of imid and ustekinumab from the models because of the non-convergence of the models

SUPPLEMENTAL TABLES- Individual lab results

Table S3b: Flamand Lab: Generalized Estimating Equation Logistic Regression, Crude and adjusted Odds Ratios (Ordinal Scores)

Characteristics	Ominicron BA1 variant		Ominicron BA5 variant	
	Crude OR (95% CI)	Adjusted OR (95% CI)[a]	Crude OR (95% CI)	Adjusted OR (95% CI)[a]
Female sex	1.16 (0.36, 3.87)	1.94 (0.34, 11.2)	1.06 (0.37, 3.09)	1.74 (0.32, 10.0)
Age at sample, years	0.71 (0.41, 1.21)	0.43 (0.17, 1.01)	0.45 (0.25, 0.76)	0.26 (0.10, 0.60)
Imid type RA	0.51 (0.16, 1.53)	-	0.54 (0.19, 1.45)	-
Vaccine type : Moderna mono	1.63 (0.08, 35.44)	0.13 (0.01, 36.2)	0.66 (0.04, 11.15)	0.92 (0, 170.2)
Vaccine type : Mixed mono	2.5 (0.32, 19.66)	3.54 (0.19, 93.0)	1.26 (0.19, 8.65)	2.72 (0.2, 39.74)
Vaccine type : Mixed bivalent	2.66 (0.52, 14.24)	2.11 (0.17, 33.3)	1.95 (0.42, 9.66)	7.69 (0.71, 89.36)
Number of vaccine :Four or more	1.39 (0.03, 66.93)	22.7 (0.12, 85.3)	2.44 (0.06, 94.6)	33.9 (0.28, 623)
Number days : 121 + days	1.19 (0.24, 5.63)	0.17 (0.01, 2.51)	0.85 (0.2, 3.58)	0.51 (0.04, 7.15)
Prednisone	0.91 (0.3, 2.69)	0.87 (0.12, 5.83)	0.54 (0.19, 1.45)	0.1 (0.01, 0.68)
Anti-TNF biologic	0.12 (0.02, 0.5)	0.04 (0.01, 0.27)	0.31 (0.08, 1.07)	0.06 (0.01, 0.29)
Ustekinumab	0.72 (0.05, 10.63)	-	0.4 (0.03, 4.84)	-
Other biologic	2.55 (0.46, 13.86)	1.17 (0.10, 11.9)	1.77 (0.29, 12.4)	0.74 (0.07, 7.88)
Methotrexate	0.42 (0.13, 1.29)	0.41 (0.05, 3.14)	0.42 (0.15, 1.17)	2.02 (0.27, 17.1)
Jak inhibitor	2.55 (0.46, 13.86)	2.52 (0.08, 88.9)	1.32 (0.25, 7.29)	0.23 (0.01, 9.3)
Other immuno	3.66 (1.05, 13.54)	3.94 (0.44, 39.5)	2.33 (0.73, 7.77)	2.39 (0.28, 22.7)
Past infection in the 6 months before sample	1.14 (0.35, 3.67)	0.24 (0.03, 1.73)	3.77 (1.24, 12.35)	1.68 (0.23, 12.6)

[a] Adjusted for all variables shown.. [c] Other biologic included : abatacept, Vedolizumab , Tocilizumab and Secukinumab.. [d] Other immuno included : azathioprine, 6-mercaptopurine, leflunomide, and sulfasalazine. Here the reference for the number of vaccines was 3 doses. Here the reference for IMID type was "SpA".
* Here the reference for vaccine type was "Pfizer monovalent". No one was exposed to rituximab and everyone was white.
**I removed the type of imid and ustekinumab from the multivariate models because of the non-convergence of the models.

SUPPLEMENTAL TABLES- Individual lab results

Table S4a; Card Lab: Generalized Estimating Equation Logistic Regression, Crude and adjusted Odds Ratios (Ordinal Scores)

Characteristics	Ancestral variant	
	Crude OR (95% CI)	Adjusted OR (95% CI)[a]
Female sex	1.84 (0.70, 4.89)	14.8 (2.48, 103)
Age at sample, years	0.74 (0.47, 1.14)	1.79 (0.85, 3.84)
White race_ethnicity	0.17 (0.01, 1.27)	0.08 (0.01, 0.97)
Imid type IBD	4.77 (0.59, 45.8)	2.84 (0.21, 44.3)
Imid type RA	4.21 (0.58, 37.0)	7.00 (0.82, 73.6)
Vaccine type : Mixed mono	1.33 (0.48, 3.79)	4.42 (0.99, 22.1)
Number of vaccine :Three or more	1.71 (0.17, 17.7)	2.09 (0.12, 35.6)
Number days : 121 + days	1.68 (0.38, 8.90)	6.24 (0.85, 51.7)
Prednisone	0.14 (0.03, 0.63)	0.07 (0.01, 0.57)
Anti-TNF biologic	0.74 (0.3, 1.84)	0.29 (0.07, 1.15)
Ustekinumab	1.07 (0.21, 6.09)	5.84 (0.46, 109)
Rituximab	0.29 (0.05, 1.53)	3.05 (0.32, 32.4)
Other biologic	8.18 (1.24, 161)	-
Methotrexate	0.20 (0.08, 0.52)	0.04 (0.01, 0.22)
Jak inhibitor	2.44 (0.43, 18.9)	2.63 (0.25, 33.4)
Other immuno	1.05 (0.42, 2.66)	2.71 (0.72, 10.9)

[a] Adjusted for all variables shown.. [c] Other biologic included : abatacept, Vedolizumab , Tocilizumab and Secukinumab.. [d] Other immuno included : azathioprine, 6-mercaptopurine, leflunomide, and sulfasalazine. Here the reference for the number of vaccines was 2 doses. Here the reference for IMID type was "SpA". Here the reference for vaccine type was "Pfizer monovalent". No bivalent from Winnipeg data.

SUPPLEMENTAL TABLES- Individual lab results

Table S4b; Card Lab: Generalized Estimating Equation Logistic Regression, Crude and adjusted Odds Ratios (Ordinal Scores)

Characteristics	Omicron BA1 variant		BA5 variant	
	Crude OR (95% CI)	Adjusted OR (95% CI)[a]	Crude OR (95% CI)	Adjusted OR (95% CI)[a]
Female sex	0.77 (0.21, 3.25)	0.57 (0.03, 7.57)	1.4 (0.51, 4.06)	6.15 (0.93, 50.26)
Age at sample, years	1.11 (0.59, 2.33)	2.02 (0.60, 11.69)	0.58 (0.37, 0.9)	0.61 (0.23, 1.45)
White race_ethnicity	0.63 (0.08, 13.16)	0.08 (0.01, 3.53)	0.2 (0.03, 1.07)	0.10 (0.01, 0.96)
Imid type IBD	0.62 (0.06, 14.63)	0.04 (0.01, 7.08)	2.71 (0.25, 62.78)	9.26 (0.29, 530.3)
Imid type RA	0.37 (0.04, 8.26)	0.15 (0.01, 8.28)	1.51 (0.15, 33.42)	1.97 (0.15, 56.77)
Vaccine type : Mixed mono	1.53 (0.36, 5.79)	0.5 (0.02, 4.73)	2.34 (0.86, 6.5)	4.32 (0.98, 21.18)
Number of vaccine :Three or more	0.24 (0.04, 1.96)	0.01 (0.01, 0.56)	0.71 (0.1, 6.05)	3.53 (0.19, 125.3)
Number days : 121 + days	4.19 (0.51, 26.7)	7.76 (0.5, 149.3)	4.04 (0.73, 23.82)	3.97 (0.49, 33.19)
Prednisone	0.14 (0.02, 0.79)	0.09 (0.01, 0.69)	0.51 (0.07, 2.55)	0.49 (0.01, 10.33)
Anti-TNF biologic	0.81 (0.16, 3.15)	0.59 (0.03, 6.79)	0.47 (0.15, 1.29)	0.08 (0.01, 0.39)
Ustekinumab	1.59 (0.08, 12.58)	2.4 (0.07, 57.92)	0.95 (0.12, 5.89)	0.21 (0.01, 5.29)
Other biologic	3.09 (0.39, 17.73)	2.18 (0.09, 59.35)	1.9 (0.32, 10.55)	14.5 (1.11, 232.9)
Methotrexate	0.69 (0.19, 2.65)	0.11 (0.01, 1.44)	0.28 (0.1, 0.73)	0.5 (0.09, 2.67)
Jak inhibitor	1.59 (0.08, 12.58)	5.18 (0.06, 309.6)	1.41 (0.17, 9.64)	0.5 (0.02, 8.49)
Other immuno	0.88 (0.18, 3.44)	1.00 (0.09, 8.65)	0.93 (0.33, 2.54)	1.42 (0.31, 6.54)

[a] Adjusted for all variables shown.. [c] Other biologic included : abatacept, Vedolizumab , Tocilizumab and Secukinumab.. [d] Other immuno included : azathioprine, 6-mercaptopurine, leflunomide, and sulfasalazine. Here the reference for the number of vaccines was 2 doses. Here the reference for IMID type was "SpA". Here the reference for vaccine type was "Pfizer monovalent". No bivalent from Winnipeg data.

SUPPLEMENTAL TABLES- Individual lab results

SUPPLEMENTAL TABLES- Individual lab results

Table S5a; Bowdish Lab: Generalized Estimating Equation Logistic Regression, Crude and adjusted Odds Ratios (Ordinal Scores)

Characteristics	Ancestral variant		Omicron BA1 variant	
	Crude OR (95% CI)	Adjusted OR (95% CI)[a]	Crude OR (95% CI)	Adjusted OR (95% CI)[a]
Female sex	4.21 (1.25, 14.68)	5.98 (1.24, 32.8)	3.77 (1.18, 13.48)	8.2 (1.49, 58.62)
Age at sample, years	0.69 (0.43, 1.07)	0.84 (0.44, 1.63)	0.72 (0.47, 1.09)	0.74 (0.39, 1.4)
White race_ethnicity	0.66 (0.14, 3.36)	0.98 (0.1, 9.47)	0.53 (0.11, 2.41)	0.84 (0.10, 7.05)
Imid type RA	0.24 (0.02, 3.74)	0.01 (0.001, 0.41)	0.2 (0.01, 2.55)	0.02 (0.01, 0.59)
Vaccine type : Moderna mono	1.25 (0.14, 9.82)	3.93 (0.28, 60.5)	0.78 (0.11, 5.18)	0.71 (0.06, 9.43)
Vaccine type : Mixed mono	0.41 (0.11, 1.49)	0.48 (0.10, 2.32)	1.12 (0.34, 3.69)	1.67 (0.36, 8.2)
Vaccine type : Mixed bivalent	0.63 (0.2, 1.99)	0.73 (0.19, 2.81)	1.25 (0.43, 3.62)	0.81 (0.2, 3.31)
Number of vaccine :Four or more	2.14 (0.85, 5.56)	2.41 (0.81, 7.47)	3.12 (1.32, 7.77)	3.61 (1.24, 11.3)
Number days : 121 + days	0.88 (0.06, 12.36)	0.35 (0.01, 9.26)	5.11 (0.39, 73.75)	5.09 (0.24, 121)
Prednisone	0.98 (0.36, 2.66)	0.85 (0.23, 3.13)	1.04 (0.43, 2.55)	0.65 (0.18, 2.24)
Anti-TNF biologic	1.27 (0.47, 3.46)	1.53 (0.4, 6.05)	0.52 (0.21, 1.26)	0.29 (0.07, 1.08)
Other biologic	0.25 (0.08, 0.73)	0.09 (0.02, 0.34)	0.3 (0.11, 0.77)	0.07 (0.02, 0.25)
Methotrexate	1.71 (0.69, 4.31)	2.55 (0.77, 8.99)	1.49 (0.66, 3.4)	0.8 (0.26, 2.44)
Jak inhibitor	0.6 (0.16, 2.2)	0.42 (0.07, 2.53)	0.42 (0.13, 1.35)	0.22 (0.04, 1.07)
Other immuno	0.96 (0.4, 2.32)	2.76 (0.76, 10.6)	0.96 (0.44, 2.10)	1.09 (0.3, 3.93)
Past infection in the 6 months before sample	1.69 (0.28, 9.36)	0.88 (0.05, 15.9)	5.73 (1.07, 35.55)	4.46 (0.3, 70.26)

[a] Adjusted for all variables shown.. [c] Other biologic included : abatacept, Vedolizumab , Tocilizumab and Secukinumab.. [d] Other immuno included : azathioprine, 6-mercaptopurine, leflunomide, and sulfasalazine. Here the reference for the number of vaccines was 3 doses. Here the reference for IMID type was "SpA". Here the reference for vaccine type was "Pfizer monovalent".

SUPPLEMENTAL TABLES- Individual lab results
